# Tendon Immune Regeneration: Insights on the Synergetic Role of Stem and Immune Cells during Tendon Regeneration

**DOI:** 10.3390/cells11030434

**Published:** 2022-01-27

**Authors:** Valentina Russo, Mohammad El Khatib, Giuseppe Prencipe, Maria Rita Citeroni, Melisa Faydaver, Annunziata Mauro, Paolo Berardinelli, Adrián Cerveró-Varona, Arlette A. Haidar-Montes, Maura Turriani, Oriana Di Giacinto, Marcello Raspa, Ferdinando Scavizzi, Fabrizio Bonaventura, Johannes Stöckl, Barbara Barboni

**Affiliations:** 1 Unit of Basic and Applied Sciences, Faculty of Biosciences and Agro-Food and Environmental Technologies, University of Teramo, 64100 Teramo, Italy; vrusso@unite.it (V.R.); melkhatib@unite.it (M.E.K.); mrciteroni@unite.it (M.R.C.); mfaydaver@unite.it (M.F.); amauro@unite.it (A.M.); pberardinelli@unite.it (P.B.); acerverovarona@unite.it (A.C.-V.); aahaidarmontes@unite.it (A.A.H.-M.); mturriani@unite.it (M.T.); odigiacinto@unite.it (O.D.G.); bbarboni@unite.it (B.B.); 2 National Research Council (CNR), Campus International Development (EMMA-INFRAFRONTIER-IMPC), Institute of Biochemistry and Cellular Biology (IBBC), 00015 Monterotondo Scalo, Italy; mraspa@emma.cnr.it (M.R.); fscavizzi@emma.cnr.it (F.S.); bonaventura@emma.cnr.it (F.B.); 3 Centre for Pathophysiology, Infectiology and Immunology, Institute of Immunology, Medical University of Vienna, 1090 Vienna, Austria; johannes.stoeckl@meduniwien.ac.at

**Keywords:** tendinopathies, inflammation, immune cells, immune modulators, innate immune response, adaptive immune response, stem cells, regenerative medicine

## Abstract

Tendon disorders represent a very common pathology in today’s population, and tendinopathies that account 30% of tendon-related injuries, affect yearly millions of people which in turn cause huge socioeconomic and health repercussions worldwide. Inflammation plays a prominent role in the development of tendon pathologies, and advances in understanding the underlying mechanisms during the inflammatory state have provided additional insights into its potential role in tendon disorders. Different cell compartments, in combination with secreted immune modulators, have shown to control and modulate the inflammatory response during tendinopathies. Stromal compartment represented by tenocytes has shown to display an important role in orchestrating the inflammatory response during tendon injuries due to the interplay they exhibit with the immune-sensing and infiltrating compartments, which belong to resident and recruited immune cells. The use of stem cells or their derived secretomes within the regenerative medicine field might represent synergic new therapeutical approaches that can be used to tune the reaction of immune cells within the damaged tissues. To this end, promising opportunities are headed to the stimulation of macrophages polarization towards anti-inflammatory phenotype together with the recruitment of stem cells, that possess immunomodulatory properties, able to infiltrate within the damaged tissues and improve tendinopathies resolution. Indeed, the comprehension of the interactions between tenocytes or stem cells with the immune cells might considerably modulate the immune reaction solving hence the inflammatory response and preventing fibrotic tissue formation. The purpose of this review is to compare the roles of distinct cell compartments during tendon homeostasis and injury. Furthermore, the role of immune cells in this field, as well as their interactions with stem cells and tenocytes during tendon regeneration, will be discussed to gain insights into new ways for dealing with tendinopathies.

## 1. Introduction

Tendons are strands of fibrous connective tissues with a great tensile strength that connect muscles to bones and allow force transfer [1]. Although tendon structure and characteristics let them endure different loadings, rapid changes or increasing of the loading forces acting on tendons can cause microtraumas and lead to tendinopathies and tendon ruptures. Tendons’ injuries are extremely frequent and the difficulty in their management represents a great worldwide medical, social, and economic challenge. In fact, ~30 million patients refer to musculoskeletal practitioners each year, with a forecast of over 25% over the next five years, because of the variations in life expectancy and lifestyle and the absence of an efficacious therapeutic solution [2].

Independently of the underlying cause of tendon disorders, it is generally accepted that a state of inflammation is frequently associated to such condition which is responsible for a poor prognosis. The core role of inflammation in tendinopathies [3] has also influenced the tissue pathology classification: ‘tendinitis’ and ‘tendinosis’ have been now recognized as an oversimplification, and actually tendinopathy is the best generic descriptive term for the clinical conditions in and around tendon disorders [4].

Inflammation can occur in tendons because of mechanical insults and other multifactorial co-triggering factors including genetic, epigenetic, systemic diseases, drugs, and in particular aging and metabolic diseases, that play a role in defining the severity of the pathological pattern and its resolution [5,6]. The disruption of normal homeostasis of tendon physiological system due to an inflammation state [3], often results in irreversible alterations of the native tendon extracellular matrix (ECM) architecture and mechanics [7]. Indeed, spontaneous adult tendon repair leads to scar tissue and fibrosis affecting hence the functionality of a tendon in terms of movement and strength [7]. Adhesion process represents another frequent complication that might occur in tendon lesions. Generally, adhesions develop as a result of inflammation which are caused by surgery or trauma [8]. In the case of tendinopathies, adhesion processes cause biomechanical disfunctions such as loss of gliding features, range of movement, and delayed healing [9,10,11]. An imbalance between fibrin deposition during the coagulation process and fibrin resolution driven by the fibrinolytic system is thought to be responsible for the creation of adhesions in tendons [9,11,12]. During tendon healing, mechanical loads play an important role in the prevention or promotion of adhesions’ development [13]. In detail, low mechanical force that accompanies limb immobilization has been proven to increase the likelihood of adhesion formation [14], whereas an increased mechanical load might provoke delayed healing [15].

Adult tendons have a limited natural healing potential which is ascribable to the peculiar tissue biology characterized by a low cellularity, hypo-vascularity and low tendon metabolism [7,16], and current treatments focusing on medication administration, exercise and surgical procedures frequently fail [7]. One of the principal reasons could be due to the fact that insights into inflammation processes in tendinopathy remain limited and researchers are still trying to comprehend the complex interaction between immune and somatic cells and the molecular systems involved, such as pro- and anti-inflammatory cytokines, growth factors and enzymes [17].

Until now, no successful targeted treatments have been developed, but it is generally accepted that understanding the key inflammatory pathways affecting tendon ECM regulation and homeostasis are critical in designing future targeted therapies for tendinopathy [3]. An innovative approach to blunt the pro-inflammatory response and accelerating tendon regeneration is represented by stem cells transplantation and/or by the injection of their immune-modulating secretomes [18,19,20,21]. Indeed, cell-based therapy and tissue engineering approaches are now being studied as promising approaches to manage the immune cells response and avoid the procrastination of the inflammatory condition to treat tendon disorders. Although, to efficaciously reach this target, it is crucial to understand how the interactions between immune cells and stem cells would impact subsequent host responses.

According to these premises, this review aims to compare the role of different tendon cell compartments in tendon homeostasis and during tendon injuries. Moreover, the role of immune cells in this field and their crosstalk with tenocytes and stem cells during tendon regeneration will be discussed to acquire insights on novel strategies applied to deal with tendon inflammatory state. It is of great importance to evaluate the interplay between the injured/healing tendon and the immune system as a model to study and better understand inflammatory processes and in particular understanding this special constellation to further expand the knowledge about other inflammatory diseases.

## 2. Tendon Structure and Homeostasis

Tendon by its nature is exposed to many mechanical loads throughout the whole life. It is characterized by a hierarchical and anisotropic structure, which allows the tendon to perform its mechanical function without breaking down. This feature is thought to be the reason due to which tendons evolve low demand for cellular and matrix turnover [22]. Collagen fibril, made up of a triple helix of polypeptides, is considered the smallest tendon functional unit that allows the tendons to exert their mechanical functions [23,24]. Tendon function depends strongly on collagen elongation and orientation. During elongation, the helix may lengthen, leading to expanding the gaps between adjoining collagen molecules, or slipping them between laterally adjacent molecules [22].

**Figure 1:**
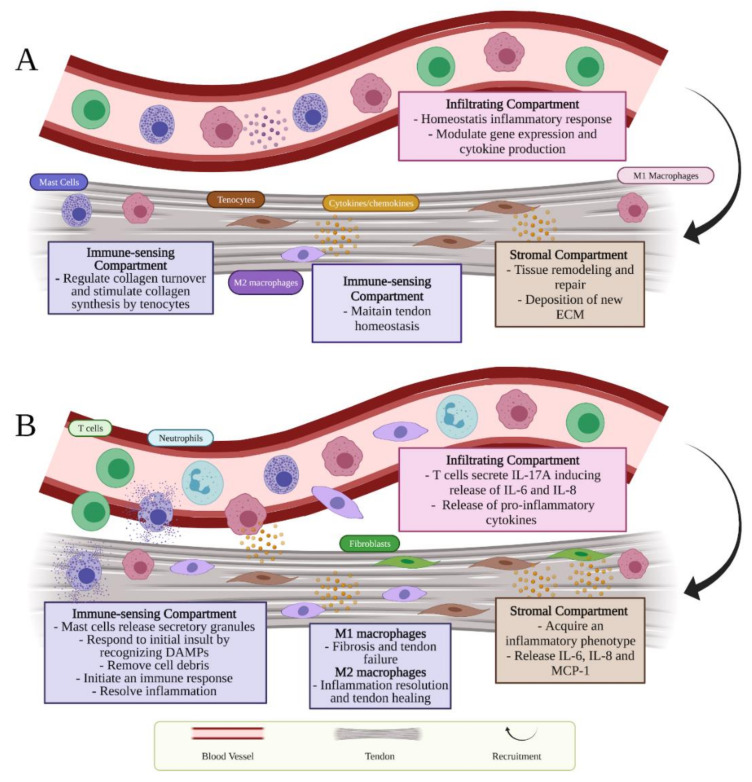
Schematic presentation of tendon immunobiology which describes the different role of cell compartments during (**A**) homeostasis and (**B**) inflammation.

Stromal, immune-sensing, and infiltrating compartments represent three different cell compartments that contribute each to a complex milieu of tendon homeostasis (Figure 1A) [3].

### 2.1. Tendon Stromal Compartment

The stromal compartment includes matrix components (i.e., collagen, ECM proteins, interfascicular matrix and small leucine-rich proteoglycans), tenoblasts, and mostly tenocytes. Recent studies have highlighted the concept of tendon cells heterogeneity [24,25,26,27,28,29] and to date, these cells cannot be uniquely identified by a specific marker [30]. Tenocytes and tenoblasts, that represent around the 90–95% of tendon cell population in mature tendons, can be mainly distinguished on the basis of their different shapes [7,30]. Tenocytes are fibroblast-like spindle-shaped cells with elongated nuclei and thin cytoplasm, while tenoblasts, immature tenocytes predominant in young tendons and mostly observed in the endotenon, are approximately round cells with ovoid nuclei [31,32,33,34]. In tendon tissue of various species, resident tendon stem/progenitor cells (TSPCs) have been also recently identified [35,36,37]. TSPCs constitute 1–4% of tendon resident cells which express positivity to CD44 and exhibit similar characteristics to mesenchymal stem cells (MSCs) [35,38]. Moreover, the concept of tenocytes heterogeneity has been recently explored further. For example, De Micheli et al. isolated Achilles tendons from C57BL/6J mice, and using single-cell RNA sequencing they identified 11 different types of cells, including 3 new populations of tenocytes (tendon fibroblasts 1 and 2, and junctional fibroblasts), according to relatively different expression of ECM proteins, including COL1A1 which is expressed at a moderate to high level [25]. Furthermore, Luesma et al. recently explored tendon tissue heterogeneity showing the presence of telocytes in the equine inter-fascicular tendon matrix near blood vessels. Telocytes could represent a subpopulation of mesenchymal progenitor cells and a source of tenoblasts [26]. In fact, it has been demonstrated that telocytes express stem cell markers such as CD34 and CD44 and it has been found to be similarly localized near the tendon stem cell niche [26,39]. This tenocyte heterogeneity, as well as the relationships between distinct cell types in tendon tissue, must be understood. Indeed, broaden the knowledge on these aspects will help researchers to better understand how tendon tissues are maintained and repaired whether during homeostasis and tendinopathies. Moreover, evolving new studies to find specific tendon cells markers will contribute to acquiring a clear view of the physiological differences between these distinct tendon cells populations.

Tissue-resident tenocytes sense mechanical stimuli and translate them into molecular response. This leads to the modulation of ECM synthesis and its remodeling due to the changes in protein and gene expression and brings the tendon to its new homeostatic setpoints, allowing it to adapt to its function within the new environment (Figure 1A) [22]. Tenocytes together with the molecules present in tendon ECM, may respond to strategies that address new mechanical and biomechanical needs of the micro-environment [22]. Latest research concerning the molecular mechanisms of tendon homeostasis has shown several signaling pathways and feedback loops demonstrating hence that the cellular response in tendon exhibits a pivotal role in preserving homeostasis. Feedback loop is the main signaling pathway for tendon homeostasis that allows the regulation of turnover ECM levels [22].

### 2.2. Tendon Immune-Sensing Compartment

Resident cells of the tendon immune-sensing compartment express immune-cell-related markers (i.e., CX3CR1, CD68, CD163) [40]. They comprise tendon-resident immune cells such as tissue-resident macrophages (M) and mast cells (Figure 1A) [3]. Tissue-resident macrophages, classified as M2 (i.e., CD163+, CD206+), have shown to exhibit a role in maintaining tendon homeostasis and resolving inflammation [41]. Moreover, Lehner et al. recently identified in healthy rodent and human tendon tissue, the presence of macrophage-like tendon-resident cells “tenophages”, which express immune cell markers including CX3CR1, CD68 and CD163. These newly identified cell population potentially accomplish a surveillance function, being activated in case of tendon tissue injury or stress, and contribute in the maintenance of tissue homeostasis [40]. Homeostatic and adaptive response are also modulated by resident mast cells, oval or irregularly shaped immune cells of the myeloid lineage, mostly identifiable by CD63 and CD203 surface markers [42]. These cells can be found in the tendon tissue or within the connective tissue nearby the paratenon, and both muscle-tendon and bone-tendon junctions and that characteristically contain 50–200 secretory granules that store inflammatory mediators [43,44]. In tendon tissue, they exhibit a role in regulating collagen turnover due the fact that these cells secrete TGF-β and fibroblast growth factor (FGF-2), which in turn stimulate the production of collagen by the tenocytes [45].

### 2.3. Infiltrating Compartment

Finally, the infiltrating compartment cells are recruited through the activation of stromal and resident immune cells that, in normal circumstances, represents a homeostatic inflammatory response (Figure 1A) [3].

Tendon homeostasis is achieved by a continuous remodeling process facilitated by matrix metalloproteases (MMPs) and matrix metalloprotease inhibitors (TIMPs), which involves the deposition by tenocytes, belonging to the stromal compartment, of new ECM, including the synthesis of collagen type I (COL1), decorin, biglycan, versican, scleraxis (Scx), tenascin C, and aggrecan (Figure 1A) [7,46,47]. Tenocytes may be stimulated through various mechanisms by applying different mechanical signals such as tension, compression, shear, and hydrostatic pressure to tenocytes [22]. Tension is the most common load that tendons are subjected to. It represents a crucial factor for preserving homeostasis. A certain level of loading is needed to maintain homeostasis and unloading can negatively affect tendon function [22]. In particular, mechanical stimuli can also activate a physiological response involving a complicated network of pathways connecting the components of cell surface which include ion channels, focal adhesion kinases, integrins, and cytoskeleton to the nucleus [48]. Collagen synthesis is noticed to augment within tendons subjected to high mechanical loads while reduced loads is accompanied by a decrease in collagen synthesis [22]. Moreover, the tendon transcriptor factor Scx, involved in tendon development and ECM synthesis, has been noticed to increase in loaded tendons while decrease in unloaded ones correlated to the reduction in ECM synthesis [49]. Additionally to Scx, Early Growth Response-1 (EGR1), a zinc finger transcription factor, has been recognized to be involved in both pre- and post-natal tendon formation [50,51,52]. Even if EGR1 is not specific for tendons, it has been demonstrated its ability in promoting sufficiently tendon gene expression such as Scx and COL1A1, during development [52]. Although its role in tendon development, transforming growth factor-β (TGF-β) has shown to be also an essential factor in mature tendon homeostasis since it induces the expression of Mohawk (Mkx) [53] and Scx [54] which in turn promote collagen synthesis [55,56]. TGF-β is active at all stages of tendon healing in which it showed to be upregulated in differentiated tendon cells [57,58,59]. TGF-β promotes collagen formation, controls proteinases and cell proliferation, and induces extrinsic cell migration. Additionally, the pathway of TGF-β expression in the human tendon is critical in the adaptability of tendon to mechanical loading [60]. TGF-β stimulates extrinsic cell migration, controls proteinases and cell proliferation, and induces collagen production. In addition, in the human tendon, the expression pathway of TGF-β is critical for tendon’s adaptability to mechanical strain [60]. It has been demonstrated that while TGF-β-Scx pathway plays a vital role in the early stage of tendon differentiation, TGF-β-Mkx pathway exhibits an essential role during the tendon maturation stage [61]. On the other hand, it has been demonstrated that individuals who spend almost their time sedentary showed increased levels of pro-inflammatory cytokines including tumor necrosis factor-α (TNF-α), interleukin-1β (IL-1β) and vascular endothelial growth factor (VEGF) accompanied whit a low level of COL1 favoring hence the state of inflammation and activating MMPs belong to MMP-2, MMP-9 and MMP-13, which may lead to an elevated risk of tendon rupture [62]. Moreover, it has been demonstrated that macrophage phenotype alters TGF-β expression levels, showing an increase on its production by M2 polarized macrophages [63,64,65,66,67,68] and TGF-β inhibition by M1 macrophages [69,70]. It has been demonstrated that priming molecules such as platelet lysate or IL-1R, that induced a polarization towards anti-inflammatory M2 phenotype, promoted TGF-β up-regulation [63,64]. In particular, Scopelliti et al. observed a significant up-regulation of TGF-β after pro-inflammatory M1 macrophages were polarized into M2 phenotype through a preconditioning with platelet lysate [63]. On the other hand, TGF-β was inhibited when M2 macrophages were primed either with miRNA-33 or miRNA-130 enriched-exosomes switching towards their pro-inflammatory M1 macrophages [67,68]. Oishi et al. [69] have also shown a higher TGF-β gene expression induced by M2 in comparison with M1 macrophages [69]. Moreover, other studies have shown lower concentration levels of TGF-β in M1 compared with M0 macrophages, suggesting the inhibitory effect of TGF-β expression by the pro-inflammatory phenotype [69,70]. Hence, these findings might indicate that uniquely the anti-inflammatory M2 macrophages have a stimulatory effect on this molecule.

## 3. Inflammatory Response Occurring during Tendon Injuries

During tissue injury, the activation of inflammatory mechanisms and the innate immune system is evident within the tendon matrix microenvironment and probably assist in dysregulated homeostasis. The three cellular compartments participating in tendon homeostasis, listed in the previous paragraph, contribute each to a complex milieu of inflammatory mechanisms during tendinopathies (Figure 1B). After tendon injury, the distinguishment between reparative versus inflammatory healing is affected by crossroads that interact between the changes occurred within the tissue microenvironment and the activation of the innate immune system.

The influential stromal compartment that contains the centrally resident tenocytes is accountable for tissue remodeling and repair. Due to the presence of immune receptors on their cell-surface, resident tenocytes secrete cytokines and chemokines in both autocrine or paracrine manner and can be driven toward an activated inflammatory phenotype (Figure 1B) [71]. The immune-sensing cells react during tendon inflammation as sentinels to respond to initial tissue insult through damage-associated molecular patterns (DAMPs) (Table 1). Alarmins represent a category of DAMPs proteins, including high-mobility group box protein 1 (HMGB1), heat shock proteins (HSPs) and S100 proteins, involved in tendon inflammation and promptly secreted into the ECM during tendinopathies [72]. HMGB1 can be released whether from the dying cells or activated stromal and immune cells and acts thanks to receptor for advanced glycation endproducts (RAGE), toll-like receptor 2 (TLR-2), TLR-4, TLR-9 and forms also heterocomplexes with IL-1ß, CXCL12 or lipopolysaccharide (LPS) [72,73]. In addition, hypoxia and mechanical stress considered crucial in starting tendon injuries seem to be key factors in HMGB1 release [72]. HMGB1 induces the upregulation of pro-inflammatory cytokines and stromal cell compartments responses. These immune-sensing cells become then activated via downstream cytokine signaling and are involved in removing cell debris and initiating inflammatory response (Figure 1B) [3]. Due to their essential function in caspase-dependent apoptotic cell signaling, HSPs may be present in rat and human models of tendinopathy and could trigger cytokine and chemokine release as well as NK cell activation [74]. S100A8 and S100A9 are low-molecular-weight calcium-binding proteins that act through the TLR-4 receptor and attract T cells, neutrophils, and macrophages [74]. These last can be distinguished based on their cell surface markers, function, and cytokine release into M1 (i.e., nitric oxide synthase (iNOS+), CD68+) and M2 macrophages. While M1 macrophages, classically activated, are considered as phagocytic and pro-inflammatory, M2 macrophages, alternatively activated, possess a critical role in suppressing the inflammation by releasing anti-inflammatory cytokines such as IL-1 receptor antagonist (IL-1RA), IL-4, IL-10 and IL-13 [3,75,76,77]. Distinct stimuli can cause the production of different subtypes of M2 macrophages: M2a, M2b and M2c [78]. M2a macrophages, also known as wound-healing macrophages, are induced by IL-4 and IL-13 and express high levels of CD206, decoy IL-1 receptor (IL-1R), and C-C motif chemokine ligand 17 (CCL17), as well as secrete pro-fibrotic factors such as TGF- β and fibronectin, leading to tissue repair. M2b macrophages, also known as regulatory macrophages, exhibit high levels of CCL1 and TNF superfamily member 14 (TNFSF14). Moreover, they produce and release considerable amounts of the anti-inflammatory cytokine IL-10 and modest levels of IL-12 and control the breadth and depth of the immune and the inflammatory responses. Finally, M2c macrophages subtype, stimulated by IL-10 via the activating signal transducer and activator of transcription 3 (STAT3), exhibit anti-inflammatory and pro-fibrotic activity by secreting high levels of IL-10 and TGF- β. In addition, M2c macrophages express high levels of Mer receptor tyrosine kinase (MerTK), involved in the phagocytosis of apoptotic cells [78]. The aberrant activation of stromal and resident-immune cells during tendinopathy contributes probably to the recruitment of infiltrating compartment cells, which include an influx of T cells, mast cells, and macrophages within the damaged area (Figure 1B) [3].

The interplay between the inflammatory response and the ECM in diseased tendons is affected by these cell compartments. In addition, the interactions between resident/infiltrating immune cells and resident stromal cells exhibit central roles in switching a spontaneously resolving inflammatory response into a chronic disease with ultimate tissue degeneration within tendon tissue [3]. Indeed, when the number of inflammatory cells becomes significant, there is an imbalance between pro-inflammatory factors with the degradation of ECM [46]. Endogenous agents produced by tenocytes and infiltrating immune cells provoke inflammation due to the activation of inflammatory mediators’ pathways, comprising cytokines (e.g., TNF-α, IL-1-β, IL-6) and prostaglandins (PGE_2_), promoting pro-inflammatory macrophage (M1) and T cell activity. These inflammatory cytokines have some effects, including the up-regulation of VEGF production together with the enhancement of the synthesis of MMPs, including MMP-1 MMP-3, MMP-8, MMP-9, and MMP-13, whose role is inducing matrix destruction. They alter ECM homeostasis, foster remodeling, increase biomechanical adaptiveness, and stimulate tenocyte apoptosis [79]. For instance, proline-glycine-proline (PGP) tripeptide represents the smallest sequence released from collagen under the action of MMP-8 and MMP-9 together with the serine protease prolyl endopeptidase. The signaling induced by PGP represents a feed-forward inflammatory signal in which their binding to CXCR1 and CXCR2 triggers the influx of neutrophils within the damaged tissues and drives them to release MMP-9 inducing the cleavage of more collagen and causing the liberation of further PGP and subsequent influx of neutrophils [79,80].

Indeed, there is a strong interplay concerning tendon ECM production and cytokines, catabolic mediators as Cyclooxygenase 2 (COX-2), PGE_2_, VEGF, and nitric oxide (NO) [17]. These complicated interactions can either impede or promote healing and restoration. Patients with tendinopathy frequently have new vessel development, usually followed by neural ingrowth [17]. It could be caused by hypoxia, which occurs frequently in metabolic illnesses and in exercise. VEGF, secreted by macrophages, has a pro-inflammatory effect additionally to other mechanisms by which tendons might be damaged, such as the over-expression of MMPs, and TIMP downregulation [17].

Evidence suggests that tenocytes, belong to resident stromal compartment, are involved in the switch from an acute to chronic inflammation [81]. The changes in the tendon microenvironment, occurred during inflammation, allow tenocytes to acquire an inflammatory phenotype by which they displayed an activated state and demonstrate their capabilities for inflammation memory [82,83]. In a series of studies conducted by Dakin et al. [83,84,85,86], they demonstrated that the markers belonging to the activated stromal fibroblasts were upregulated in the diseased human tendons compared to the healthy ones. Moreover, stromal cells collected from diseased supraspinatus human tendons have shown an elevated gene expression of interferons (IFN) and nuclear factor-kappa B (NF-κB) after being treated with lipopolysaccharides or IFNγ when compared to stromal cells belong to healthy tendons [84]. Additionally, it has been proved that the incubation of stromal cells collected from diseased and healthy human tendons in specialized pro-resolving lipid mediators (SPM) stimulated the synthesis of more pro-resolving mediators thereby suppressing the expression of pro-inflammatory molecules such as PGE_2_, IL-6, signal transducer and activator of transcription 1 (STAT-1), and podoplanin (PDPN) [85]. In another study conducted by Stolk et al. [71], the authors demonstrated that tenocytes derived from human ruptured supraspinatus tendon altered their surface markers and boost the secretion of IL-6, IL-8 and monocyte chemoattractant protein 1 (MCP-1) when being stimulated with activated mononuclear cells [71]. These findings imply that the insistent activation of stromal fibroblasts is implied in the development of chronic inflammatory tendinopathy [71].

Moreover, when immune-sensing cells are overstimulated, tissue breakdown and degeneration occur [87]. The activated mast cells react by their degranulation allowing the release of secretory granules composed of proteoglycans (serglycin), mast cell specific proteases (chymase, tryptase, and carboxypeptidase A3: CPA3), non-mast cell specific proteases (MMP-9, active caspass 3, renin), biogenic amines (histamine, serotonin, dopamine, and polyamines), lysosomal enzymes (β-hexosaminidase, arylsulphatase A, cathepsin C), growth factors and certain cytokines such as TNF-α, IL-4, TFG-β, IL-5, IL-6, and VEGF [45,88]. It has been demonstrated that some molecules secreted by activated mast cells could greatly affect both inflammatory and proliferative phases after tendon injury. For example, neo-angiogenesis and nerve ingrowth might be affected by VEGF and nerve growth factor (NGF). Although its contributing role in collagen synthesis, mast cells might assist in the degradation of collagen due to the activity of released proteases [45].

Infiltrating-immune cells exhibited critical role during inflammation. In a study conducted by Marsolais et al., they induced an injury in Achilles tendons in rats to assess the time course of immune cell accumulation [89]. They found that tendon defect is accompanied by a classic immune cell infiltration characterized by an increased neutrophil and CD68+ macrophage population at day 1, followed by their decrease at 7 and 14 days post-injury and accompanied by the increase of CD163+ macrophages after 28 days [89]. Additionally, it has been highlighted that infiltrating leukocytes exert a key role in tendon disease [5,90]. It has been suggested that T cells and innate lymphocytes are beyond the secretion of IL-17A, a pro-inflammatory cytokine, found in early human rotator cuff tendinopathies [90]. The study conducted by Millar et al. demonstrates that IL-17A might induce the release of other inflammatory cytokines (IL-6 and IL-8), which in turn affect the reparation-degeneration balance [90].

Moreover, supporting tissues including vascular and nervous ones play an important role in modulating the inflammatory response of the injured tissue. The presence of neural sprouting with angiogenesis is significant when assessing the role of inflammation [91]. In addition, the neurological system is significant in tendon homeostasis and pain modulation. Indeed, opioids, neuroregulators, autonomous, and excitatory glutamatergic neurotransmitters, operate in neuronal control in healthy and damaged tendons through the modulation of cell proliferation, cytokines and growth factors expression, immunological responses, and hormone release [92]. ‘Neurogenic inflammation’—the release of pro-inflammatory mediators such as Substance P, calcitonin gene-related peptide (CGRP), and calcitonin and endothelin—might assist to the progression and the pain of tendinopathy [91]. However, the neuronal dysregulation in tendinopathy, characterized by an excess in sensory and glutamatergic neurotransmitters and for a long time, is thought to trigger pain signaling and hyper-proliferative/degenerative events, followed by abnormal augmentation of sprouting sensory nerves and Substance P expression [93,94].

In addition, tendinopathies are multifactorial diseases where aging and simultaneous metabolic diseases may have a role. Advanced Glycation End-products (AGEs) belong to other endogenous inflammatory initiators, which are observed in aging and in Type I and Type II Diabetes. The precise mechanism by which these endogenous factors activate inflammation is still unknown. In diabetes mellitus, it is hypothesized that when blood glucose availability is elevated, the synthesis of AGEs is markedly elevated [17]. These are associated with protein degradation, nitric oxide destruction, and growth factor inhibition, augmenting apoptosis via oxidative stress and increased activity of pro-apoptotic and pro-inflammatory cytokines [17]. Moreover, it is now accepted that “inflammageing”, the age-related rise in the systemic pro-inflammatory condition, occurs in aging tendons, and that older people displayed a diminished ability to resolve this process. Therefore, aging might lead to deregulated tendon repair via IL-1β induced PGE_2_ pathways [17] and by a reduced tendon resolution response that decreases with age [95].

Tendon overuse together with the different contributing conditions that lead to the persistence of inflammation affect the tendon resolution state whether towards a regenerative or degenerative one. Indeed, the persistence of inflammatory mechanisms and of innate immune system activation within fibrotic tendon matrix microenvironment is indicative of the loss of tissue homeostasis and the interplays between the different cell compartments determine if the inflammatory response within tendon tissue follow a reparative or degenerative chronic state.

It is of great importance to build a tendinopathy targeted therapy whose role relied on the modification of pro-inflammatory response and the promotion of regeneration. Despite advances in insights into the molecular mediators implicated in tendon disease, successful focused therapeutics have eluded the research thus far. The information collected from the preclinical studies offer interesting cues to define molecular mechanisms implied in the modulation of the inflammatory response and its guidance towards an anti-inflammatory role. Thus, it is of great interest to identify highly specific molecules whose aim is to modulate the inflammatory signaling of immune cells by reprogramming their phenotype towards a pro-regenerative one together by using the immuno-modulatory paracrine factors produced by stem cells which have shown a great potential on inducing macrophage shift from a pro-inflammatory to pro-regenerative phenotype allowing a reduction in immune cells infiltration and deposition of an organized ECM [20,96]. Indeed, the crosstalk between stem and immune cells during tendon regeneration will be discussed in the paragraph 6.

Inflammation suppression has therefore been proven to ‘turn off’ the activation of important inflammation-resolving pathways. However, while the inflammatory component of tendinopathy has gotten a lot of attention, pro-resolving pathways in wounded tendons haven’t gotten nearly as much attention. Employing the potential of pro-resolving mediators, to lead the resolution of tendon inflammation, represents a new way to lead the resolution of tendon inflammation, rather than just passive cessation of inflammation [97]. The discovery of specific pro-resolving mediators (SPM), which include lipoxins such as lipoxin A 4 (LXA 4) that operate on the formyl peptide receptor 2 (FPR2/ALX), adds an exciting new aspect to inflammation [95]. Indeed, during the early stages of tendon damage, Dakin et al. found an increased expression of the pro-resolving receptor FPR2/ALX, as well as a transition from pro-inflammatory prostaglandins to pro-resolving lipids synthesis such as LXA 4 [97]. Moreover, FPR2/ALX is expressed by monocytes and macrophages and is important for limiting the length and amplitude of the inflammatory response by giving endogenous stop signals. Therefore, resolution pathways are pre-programmed responses that are engaged during inflammation, promoting hence inflammation resolution and tissue restoration to its normal homeostatic state. Furthermore, phagocytosis of apoptotic cells, modification of inflammatory cell infiltration to the inflamed region, and manipulation of vascular permeability are all part of the inflammation resolution process [95]. Thus, understanding the key inflammatory pathways influencing tendon ECM modulation and homeostasis is crucial when developing future targeted therapeutics for tendinopathy. Understanding these pathways may aid in defining the molecular checkpoints that direct a homeostatic inflammatory response toward an abnormal inflammatory tendon milieu that leads to clinical tendinopathy.

## 4. Crosstalk between Tenocytes and Immune Cells during Tendon Regeneration

**Figure 2:**
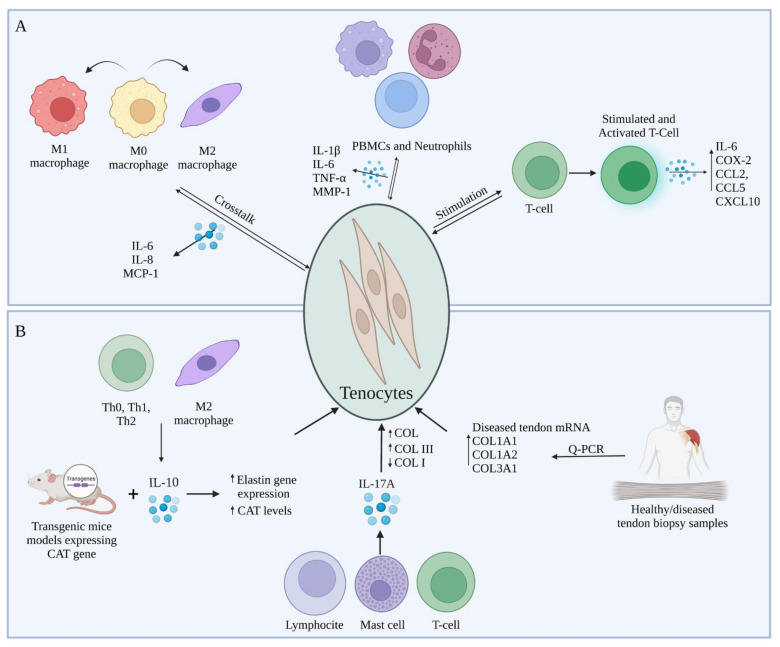
Representation of the prevalent immunomodulatory crosstalk between tenocytes and immune cells during tendon healing tested (**A**) in vitro and (**B**) in vivo.

As in depth described in the previous paragraph, platelets, polymorphonuclear leukocytes, macrophages, and other inflammatory cells belonging to the immune-sensing or infiltrating compartments produce growth factors and cytokines in response to wounding and inflammation [7]. In return, tenocytes which represent the stromal compartment interact with these immune cells in an inflammatory environment, thus influencing the surface markers of both compartments, as well as cytokine and collagen synthesis [71]. In fact, the research performed in the last decade have demonstrated the important role that immune cells play in tendinopathies, taking into account their interactions with tenocytes [71,84,98]. Specifically, tenocytes do respond to pro-inflammatory cytokines stimulation and other signals released from immune cells already present or recruited at the injury site by switching their phenotype towards an activated inflammatory one, thus altering the expression of their surface markers and secreting cytokines and chemokines [84,99] such as IL-6 and MCP-1, respectively [71,84,98]. Additionally, tenocytes are thought to multiply and become more metabolically active in response to inflammatory cytokines and growth factors including the platelet-derived growth factor (PDGF), insulin-like growth factor 1 (IGF-1), and TGF-β, which in turn can shift towards hyperplasia and hypertrophy [100]. Going more in depth in the crosstalk between tenocytes and immune cells as well as identifying the key molecular factors involved in vitro (Figure 2A) and in vivo (Figure 2B) might help to comprehend the processes that delay immunological and therapeutical resolution following tendon damage [71,101,102,103].

In vitro studies have concentrated on the effects of immune cell-released cytokines on the expression of tendon ECM genes and proteins in healthy, diseased, and damaged human and animal tendons, as well as their subsequent effects on tenocytes. In this context, several cytokines have been proposed to be involved in the inflammatory response after tendon injury, in particular IL-1β, IL-6, and TNF-α being the most studied in tendinopathies [104]. Al-Sadi et al. demonstrated, in vitro, the presence of a soluble mediator-dependent relationship between peripheral blood mononuclear cells (PBMCs) and neutrophils with tenocytes, that results in the production of numerous pro-inflammatory cytokines, out of which IL-1β, TNF-α, and IL-6, and other substances, including MMP-1 [98]. These soluble compounds exhibit multiple roles in the mechanism of the post-injury reaction inside the tendon. Indeed, several researchers described these biomolecules as stimulators that initiate inflammation, cell death and ECM degradation within damaged tendons [71,105]. Moreover, in a research performed on tenocytes exposed to IL-1β, a pro-inflammatory cytokine essential for the organism response and cell recruitment [106], it has been demonstrated the presence of catabolic effects such as enhanced matrix MMPs production [104] and that tenocytes previously activated with IL-1β, in vitro, can promote the migration of activated T cells by upregulating intercellular adhesion molecule 1 (ICAM-1), a gene involved in the inflammation and T cell recruitment [101]. In a study conducted by Garcia-Melchor et al., the authors demonstrated that the direct interaction between T cells and tenocytes contributes to further stimulation of T cells, which in turn upregulate the expression of inflammatory messenger molecules represented by an increase in IL-6, IL-8, COX-2, CCL2, CCL5 and CXCL10 gene expression [101]. Furthermore, the T cell activation increased the ratio between COL3 and COL1 in the stromal compartment, forming an interplay that could contribute to the establishment of a chronic inflammatory response, accompanied by changes in biomechanical properties of the tendons [101]. Interestingly, two co-culture systems were used within the same study to determine the difference between the results obtained in direct contact versus trans-well system. The obtained results demonstrated an increase in CD69 and IFN-γ expression specifically within T cells when exposed to direct contact with tenocytes as opposed to trans-well indirect contact experiments, meaning a significant increase in T cells’ activation [101]. Indeed, CD69 works as a costimulatory molecule for T cell activation and proliferation [107]. Multiple subtypes of T cells, including CD4+ and CD8+ T cells also synthesize and express IL-17 (IL-17A), a pro-inflammatory cytokine often found at the injury site [108]. Millar et al. proposed that IL-17A may serve as a cytokine modulator of early tendinopathy, describing it in in vitro studies [90]. Even though lymphoid lineages, especially T cells and innate lymphocytes, are noticeably crucial IL-17A producers, mast cells [109], macrophages [110], and neutrophils [111] may also contribute to the local cytokine pool, either by synthesis or receptor-mediated absorption and subsequent release [90]. IL-17A has a number of actions that contribute to tissue damage and disintegration during inflammation. It specifically stimulates the production of cytokines such as IL-1, IL-6, TNF-, chemokines, and MMPs [112]. During the experiments, tenocytes were shown to be less pro-inflammatory when IL-17A was inhibited [90].

The interactions of tenocytes with immune cells does not limit to lymphocytes and leukocytes but they also interact with macrophages and mast cells [71]. In the event of macrophages, tenocytes secrete molecules that change the polarization markers expression in macrophages during the early stages of inflammation and upregulate IL-6, IL-8, MCP-1 in a co-culture system [71]. In detail, in an experiment conducted by Stolk et al., they co-cultured M0 macrophages with pre-stimulated tenocytes to assess their crosstalk in vitro. The obtained results showed a significant increase in cytokine expressions under both direct cell and trans-well co-culture system conditions. Interestingly, it has been observed a significant increase in CD80 expression, simultaneously with a decrease in HLA-DR expression, which are both typical M1 polarization markers [71]. Thus, it can be supposed based on the obtained results that pre-stimulated tenocytes and macrophages co-culture induced a partial activation of macrophages. Moreover, in response to tenocyte activation, M1 macrophages start to express and release numerous pro-inflammatory cytokines, specifically TNF-α, IL-1, IL-6 and iNOS or change their phenotype towards an anti-inflammatory M2 one [113]. TNF-α presents the capabilities to affect tenocytes matrix synthesis catabolically, and activating them in other to produce cytokines and matrix-degrading enzymes [114] and also to decrease COL1 expression [114,115]. In a series of studies, Behzad et al. observed the impact of mast cells on tenocytes’ function and secretory activity [116]. The study aimed at determining the mechanism involved in the tenocytes stimulation by the mast cells inducing the release of soluble molecules that affect tissue remodeling and the biomechanical properties of the tendon [116]. Firstly, tenocytes exposed to mast cell line conditioned media presented a higher viability rate compared to the control sample during the in vitro studies [116]. Additionally, the obtained results showed that the contact with mast cells conditioned media stimulated human tenocytes to increase the levels of COX-2, which resulted in an increase in the PGE_2_ levels [117]. This upregulation of PGE_2_ resulted in a decrease of the COL1A1 mRNA levels, thus affecting the biomechanical properties of the tendon [116,118].

M2 macrophages [119] and Th0, Thl and Th2 cell subsets [120] also produce IL-10, which has a regulatory function on several cytokines, including IL-1, IL-2, IL-8 and TNF-α [121]. IL-10 can also coordinate connective tissue remodeling and suppression of the production of pro-inflammatory cytokines such as TNF-α in immune cells, due to their anti-inflammatory capabilities and their possible role in extracellular matrix remodeling [114,121]. IL-10 levels can be influenced by IL-6, other cytokine subtype produced by M1 macrophages [122,123]. It has been demonstrated that IL-6 promoted the acute phase protein synthesis as well as neutrophil formation in the bone marrow [123]. It has been proposed by Qi et al. that animals lacking the pro-inflammatory cytokine IL-6 during tendon healing express less inflammation and complete greater collagen synthesis, organization and higher material properties, and therefore, the presence of this cytokine secreted by tenocytes causing delayed healing [124].

Although many studies have been conducted to assess the crosstalk between tenocytes and different immune cells in vitro, few studies have been performed in vivo and in clinic in this regard in terms of the interaction and effect of immune cells and the soluble molecules released on the tendon and the mechanism of tendinopathy. For instance, Reitamo et al. used transgenic mice models expressing the human elastin gene chloramphenicol acetyltransferase (CAT) in order to observe the effect of IL-10 on elastin gene expression, in vivo. IL-10 was administered subcutaneously at a concentration range between 1–100 ng. They discovered that recombinant human IL-10 can upregulate elastin gene expression in vivo demonstrated by the increased levels of CAT, indicating the role that IL-10 might play in the production and degradation of extracellular matrix [121]. Jelinsky et al. and Millar et al. both conducted research on human models of tendinopathy [90,125]. The study conducted by Jelinsky et al. was performed on tendon biopsy samples which were obtained from a diverse group with variations in age, gender, symptoms, illness stage, and physical activity. The aim of the study was to determine what cytokines are upregulated or downregulated and quantify MMP and collagen in human tendinopathy samples compared to normal tendons. After analyzing the gene expressions, they have observed that mRNA levels COL1A1, COL1A2, and COL3A1 were elevated in the diseased tendon samples compared to healthy ones. Moreover, pro-inflammatory cytokine levels were not upregulated, thus confirming the hypothesis that they lack implication in the later stages of tendinopathies [125]. Millar et al. directed an experiment in which they aimed to demonstrate the role IL-17 plays in the inflammation response and tissue remodeling. Numerous torn supraspinatus human samples were examined from different age groups in patients already showing different levels of symptoms, and to compare with early stages of inflammation, biopsies were extracted from intact subscapularis muscle as well. Results have shown a difference between the levels of IL-17 mRNA levels in the suprascapularis samples as compared to supraspinatus and control group samples, which suggests the implication of IL-17 in the early stages of tendinopathies. Moreover, IL-17A not only enhanced total collagen production, but it also appeared to shift collagen production from type I to type III. Therefore, the presence of IL-17A might result in negative mechanical alterations in the extracellular matrix of the tendon [90].

Taking together, tenocytes play a crucial role in modulating the gravity of tendinopathies due to their influence on the reactions that submerge after tendon damage by communicating with immune-sensing cells, attracting, and activating the infiltrating immune cells into the injury site, or modulating the secreted implicated cytokines. These soluble molecules not only activate and boost tenocytes to proliferate, but also shift them towards an inflammatory phenotype, and thus secreting more molecules to continue the inflammation process. Understanding the crosstalk between tenocytes and immune cell compartments can allow further comprehension of the mechanisms taking place during all stages of tendinopathies to develop new strategies aim at blunting the inflammation process. Regenerative medicine represents an emerged strategy implies the use of stem cells, characterized by their anti-inflammatory properties, or their secretome, allowing the shift of tendon cell compartments towards an anti-inflammatory phenotype thus ameliorating tendon regeneration.

## 5. Crosstalk between Stem Cells and Tenocytes to Modulate Their Inflammatory Phenotype

**Figure 3:**
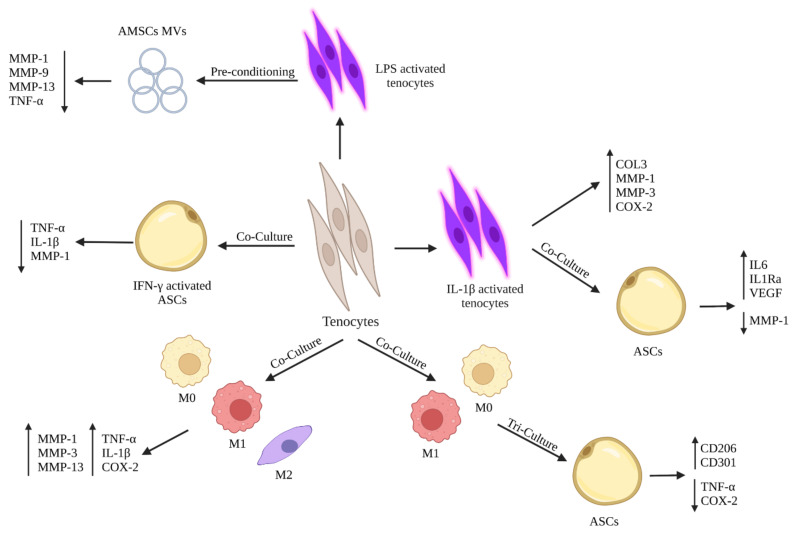
Schematic representation of the potential crosstalk between stem cells tenocytes on modulating tenocytes’ inflammatory responses in vitro.

Tenocytes, as described in paragraph 3, during tendon inflammation can be activated towards an inflammatory phenotype and start secreting chemokines and cytokines to modulate the immune response and ECM remodeling within the damaged tendons (Figure 3) [71]. Different in vitro studies have demonstrated that the treatment of tenocytes with inflammatory factors such as IL-1β or IL-6 induced a significant upregulation in matrix degradation (MMP-1, MMP-3, and MMP-13) and inflammation related factors including TNF-α, COX-2 and cytosolic phospholipase A2 (cPLA2) accompanied with a downregulation of ECM related markers [47,114,126]. To blunt the pro-inflammatory phenotype of tenocytes, novel strategies have been applied in the field of regenerative medicine by using stem cells and/or their secretomes. These strategies aim at the use of mesenchymal stem cells (MSCs), the most studied to date, for review [127,128], and amnion derived cells [18,20,129,130,131,132,133], which have, together with their derivatives including secretomes and exosomes, immunomodulatory properties able to modulate the inflammatory response in-vivo and improve tendon regeneration and ECM remodeling, as described in detail in paragraph 6 [18,19,127,128,129,134]. Thus, understating how stem cells and/or their derivatives can modulate the inflammatory phenotype of tenocytes represents a crucial aspect to be considered in tendon regeneration.

Although the existence of several publications concerning the tenogenic differentiation of stem cells by expressing tendon-related markers and their role on improving ECM deposition, few studies have concentrated on the role of stem cells and/or their derivatives on blunting tenocytes’ inflammatory phenotype. In particular, Manning et al. examined the ability of adipose-derived mesenchymal stem cells (ASCs) on modulating the inflammatory phenotype of tenocytes subjected to macrophages in a tri-culture system [135]. When tenocytes were co-cultured with three different macrophages phenotypes (M0, M1, and M2), there was an upregulation of pro-inflammatory factors (TNF-α, IL-1β, and COX-2) and matrix degradation related markers (MMP-1, MMP-3, and MMP-13) by tenocytes. Instead, when tenocytes were co-cultured with IFN-γ activated ASCs, there was a downregulation in the gene expression of TNF-α, IL-1β, and MMP-1. Thus, exposing tenocytes-macrophage co-culture to activated ASCs blunted the inflammatory phenotype of tenocytes by downregulating pro-inflammatory mediators. Moreover, tenocytes modulated their responses on M1 macrophages when cultured with ASCs [135], in which it has been noticed a downregulation of inflammatory mediators (TNF-α and COX-2) by tenocytes within the tri-culture system compared to normal co-culture between tenocytes and M1 macrophages [135]. Additionally, exposing the co-culture system between tenocytes and different macrophage phenotypes (M0, M1, and M2) to ASCs led to a change in macrophage phenotypes in which M0 and M1 macrophages showed an increase in the expression of CD206 and CD301, two cell surface marker specific for M2 macrophages [135]. Indeed, the obtained results demonstrated that ASCs could suppress the negative effects of pro-inflammatory M1 macrophages by shifting their phenotypes towards anti-inflammatory M2 macrophages. Another study conducted by Viganò et al. aimed at evaluating the ability of ASCs extracted from microfragmented adipose tissue (µFAT) to counteract the inflammatory phenotype of tenocytes stimulated by IL-1β [136]. The subjection of tenocytes to IL-1β induced an upregulation in the COL3, MMP-1, MMP-3, and COX-2. Instead, the co-culture of IL-1β activated tenocytes with ASCs resulted in a downregulation in MMP-1 with an enhanced production of IL-6, IL-1Ra, and VEGF. Thus, the paracrine action of ASCs, exercised by the secreted anti-inflammatory mediators, modulated tenocytes’ inflammatory phenotype and inhibited the expression of fibrosis and catabolic markers [136].

Other studies were not limited to the effects of stem cells on modulating the inflammatory phenotype of tenocytes, but they also evaluated the effects of stem cells’ paracrine factors including microvesicles (MVs) and conditioned media (CM) on tenocytes’ behavior. In two studies conducted by Lange-Consiglio et al., they demonstrated that the use of MVs secreted by amniotic mesenchymal stem cells (AMSCs) induced a downregulation in the expression of pro-inflammatory markers by tenocytes with no effect on the proliferation of peripheral blood mononuclear cells (PBMCs) [137,138]. In detail, Lange-Consiglio et al. studied the effect of AMSCs MVs on equine lipopolysaccharide (LPS) activated tenocytes [138]. The obtained results showed that MVs counteracted in vitro inflammation of LPS activated tenocytes by downregulating their expressions of MMP1, MMP9, MMP13, and TNFα with no effect on PBMCs proliferation, in contrast to CM [138]. The above-mentioned results demonstrated that MVs were able, due to their content of anti-inflammatory mediators, to target tenocytes and modulate their inflammatory phenotype.

Taking together, the rescue of tenocyte resident cells from acquiring an inflammatory phenotype can counteract the release of pro-inflammatory mediators within the damaged tendon tissues under the effect of stem cells and their derivatives, including MVs and CM, which in turn can start ECM synthesis and remodeling driven by tenocytes.

## 6. Crosstalk between Stem Cells and Immune Cells during Tendon Regeneration

The tissue regenerative advances provided by stem cells and/or their secretomes are to be mediated by their role in suppressing the proinflammatory response and improving the resolution of tendon disease. This novel approach could spare inflammation-induced healing moieties and stimulate robust and rapid ECM repair.

Regenerative medicine comprehends all biological therapeutic strategies to improve tissue lesion and disfunction [139]. For example, stem cells are able to promote cells homing of progenitor cells involved in regenerative process as demonstrated by the recruitment of CD45+ positive cells by ADSCs seeded on biomimetic hybrid nanocomposite scaffolds engrafted into chest defect as a chest wall graft [140]. Concerning tendon regenerative medicine, the most used strategies to improve healing and regeneration are stem cells- based therapies [141,142], injections of platelet rich-hemoderivatives (PRHd) such as platelet-rich plasma (PRP) [143,144], gene therapy [145] as well as biomaterial-based strategies [146] and TE approaches [24,147,148].

In order to define the ideal stem cell to stimulate tendon healing, in vitro protocols have been attempted to test the tenogenic plasticity of different stem cell source sometime in combination with in vivo preclinical studies [24,149]. The rationale behind the use of stem cells in regenerative medicine is to promote regeneration limiting scar tissue formation, exploiting the tendon differentiation potential of stem cells as well as by taking advantage of their paracrine influence on host tissue in orchestrating the ECM remodeling and in modulating the inflammatory reaction through the production cytokines able to modulate inflammation [150]. As stem cell therapy seems to be a promising treatment for tendon injuries and tendinopathies [151,152], investigating how transplanted stem cells interact with the inflammation and immune host cells is a crucial argument. In fact, the acute phase of tendon injury is characterized by high level of inflammation and by the presence of inflammatory cytokines such as IFN-γ, TNF-α and IL-1β [153,154].

MSCs are largely used in tendon regeneration as they have proven to have regenerative potential [155]. MSCs can be harvested from tendon tissue as tendon stem cells (TSCs) or from other tissue such as the bone marrow (BM) or the adipose tissue [96]. Their pro-regenerative abilities are due to MSCs effect on macrophages and influence on the immune response [156], inflammation and tissue repair [157]. In fact, MSCs can release cytokines that influence the macrophage polarization and function [158,159]. These cytokines released by MSC can recruit macrophages and endothelial cells to repair injuries and stimulate wound healing [160].

PGE_2_, which is generally considered a proinflammatory lipid mediator, also exhibits powerful and context-dependent anti-inflammatory effects [161]. In vitro experiments showed that MSCs can produce PGE_2_ that could be able to switch M1 macrophages into M2 or improve the selective activation of M2 [162] with anti-inflammatory properties that suppresses immune activity by interacting with natural killer (NK) cells, cytotoxic T lymphocytes (CD8+) cells and regulatory T cells (TREGS) [163]. PGE_2_ is produced through a COX-2-dependent Prostaglandin E synthase (arachidonic acid pathway) [164]. By co-culturing M1 macrophages with COX-2 knockdown (COX-2KD) MSCs, these last were incapable of attenuating the pro-inflammatory cytokine TNF-α. Moreover, COX-2KD MSCs were not able to induce M1 to M2 switch. Hence, these findings suggest that the COX-2-dependent production of PGE_2_ in MSCs plays a significant role in macrophage polarization/metabolic changes [162]. These studies are consistent with the findings of Digiacomo et al., who found that PGE_2_ synergizes with colony stimulating factor (CSF-1), resulting in CSF receptor transactivation, which supports monocyte/macrophage survival, differentiation, and motility [165]. Moreover, it has been demonstrated by Rossi et al. that PGE_2_ contained within CM, obtained from human amniotic membrane (hAM) and human amniotic MSCs (hAMSCs), is involved in the immunomodulatory activity of hAM and hAMSCs. In addition, this study revealed that the exposition of PGE_2_ to T cells induced a slight reduction in their proliferation together with an inhibition of PBMCs proliferation confirming hence its anti-inflammatory role [166].

In vivo experiments demonstrated that MSCs can influence M2 polarization also by secreting the paracrine IL-1Ra [167] and by micro-RNAs [168]. In addition, MSCs exosome can influence the immune response as C-C chemokine receptor type 2 (CCR2) positive exosomes influence CCL2 recruiting and activating macrophages [169]. Transplanted MSCs can influence T cells migration avoiding the infiltration of immune cells at the site of inflammation [170]. MSCs can recruit T cells and regulate their activation and differentiation, overexpressing chemokine (C-X-C motif) ligand 9 and 10 (CXCL9 and CXCL10, respectively), when stimulated by inflammatory factors (such as IFN-γ and TNF-α) [171]. MSCs can produce immunomodulatory factors, such as induced iNOS, indoleamine 2,3-dioxygenase (IDO), TGF-β, and PGE_2_ that inhibit T cell proliferation [172]. In fact, during inflammation, IDOs modulate the T cells response [173]. Moreover, MSCs secrete heme oxygenase-1 (HO-1) that suppresses T cells [174]. MSCs inhibit T cell activation through different pathways. For example, MSCs may secrete Fas ligand (FasL) and tumor necrosis factor-related apoptosis inducing ligand (TRAIL), which induce apoptosis and inhibit the activity of T cells [175,176]. MSCs can regulate inflammatory response enhancing Treg cell differentiation and inhibit T helper (Th) 17 by the action of their secreted factors TGF-β, PGE_2_, Notch1, and IL-10 [177]. MSCs can also influence innate T cells [159,178] as they have an impact on the level of costimulatory ligands in antigen-presenting cell (APCs) [179] needed for the activation of T cells [178]. In addition, the MSC secreted factors influence the expression of IL-12, TGF-β, IL-1 and IL-10, in APCs that control the differentiation of T cell subsets [172,180].

The immunomodulation of MSCs in tendons has not been largely investigated. However, an in vitro study on adipose derived mesenchymal stem cells (ADMSCs) demonstrated their ability to modulate the immune response during tendon inflammation [135]. In this study, mice tendon fibroblasts (TF) were cultured in different condition with and without IL-1β, macrophages (M0, M1, M2) and/or ADMSCs to test their reaction after induced inflammation and to assess the crosstalk between different cells type [135]. Results demonstrated that IL-1β had a negative effect on TF contributing to decreased viability, upregulation of inflammation related genes, matrix degradation and downregulation of ECM remodeling factors [135]. The co-culture of TF with macrophages generally demonstrated a negative effect especially caused by that M1. In TF+M1 co-culture, the addition of ADMSCs resulted in downregulation of IL-1β and TNF gene expression by TF compared to TF+M1 alone, after 5 days. On the other hand, co-culture of ADMSCSs with TF+M0 promoted their polarization towards M2 phenotype as demonstrated by the upregulation of the marker CD206 and CD301 [135]. Moreover, M2 phenotype markers increased after 1 day, when M0 cells were cultured with ADMSCSs. This study represents a valid in vitro model to study the crosstalk between resident tendon fibroblast cells, immune and stem cells, demonstrating that ADMSCSs modulated the negative effect of M1 and promoted the M0 polarization towards M2 [135].

MSC immunomodulation after transplantation has been investigated in different body districts such as bone [181] and liver [182].

Evidence reported the in vivo healing effects of untreated and TNF-α primed MSCs derived from BM in rat Achilles segmental defect model [183]. Rat Achilles tendons, subjected to a unilateral segmental defect, were repaired with either MSC or TNF-α-primed MSC seeded on poly(lactic-co-glycolic acid) (PLGA) scaffold. In vivo, both treated and untreated MSCs, augmented IL-10 production and diminished the inflammatory factor, IL-1 α. TNF-α-primed MSCs downregulate IL-12 synthesis and the number of M1 macrophages, along with increased the percent of M2 macrophages, and synthesis of the anti-inflammatory factor IL-4. The effect of BM-MSCs was also reported in ligament regeneration. In this research the right number of cells to be inoculated to have the optimum results was also studied [184]. In particular, 4 × 10^6^ cell density was compared to 1 × 10^6^. Surprisingly, the lower concentration resulted the best to induce regeneration and decrease inflammation. The high dose of MSCs resulted in increased pro-inflammatory cytokines at day 5 (IL-1β, IFN-γ, IL-2) and increased expression of IL-12 at day 14 after injury and transplantation. On day 14, M1 macrophages were present in the wounded site treated with high dose of MSCs while a sensible decrease of M2 was detected compared to controls. The low dose of MSCs resulted in a small wound size after injury and MSCs inoculation. These results, together with the significant changes in procollagen I, proliferating cells, and endothelialization suggests that MSCs can affect the cellular response during healing in a dose-dependent manner [184].

Currently the most promising stem cells source for tendon TE are the tendon stem cells (TSCs) that were discovered in several species as human, horse, rabbit, rat, and mouse [34,185,186,187] and conserved the ability to spontaneously differentiate into tenocyte [188].

However, even if TSC may represent the ideal therapeutic stem cell source, their effect is poor in term of cells availability, in vitro expansion without phenotypic drift [189] and in immunomodulatory property evidence collected to date. Nevertheless, it was demonstrated that they have low immunogenicity and allogeneic transplantation could be recommended [190]. In fact, allogenic TSCs from rat patellar tendon provoked a feeble lymphocyte proliferation, and they could escape from the lymphocyte mediated cytotoxicity [190]. Moreover, the anti-inflammatory role of TSCs was previously demonstrated by the decreased number of lymphocytes upon allogenic TSC delivered via silk–collagen scaffold for rotator cuff repair in rabbits [191].

It was proposed a role of TSCs in modulation of inflammation and matrix metalloproteinase (MMP) activities during tendon healing. TSCs treated with connective tissue growth factor (CTGF) express anti-inflammatory IL-10 and TIMP-3 through c-Jun N-terminal kinase (JNK) and signal transducer and activator of transcription 3 (STAT3) signaling, that play essential roles in tendon healing [192]. In samples inoculated with CTGF there was a significantly reduced number of iNOS^+^ M1 cells in the early healing phase was detected, while the CD146^+^ TSCs increased correlated to an increased IL-10 expression. IL-10 has a crucial anti-inflammatory role in tendon healing inhibiting the release and the action of proinflammatory cytokines and monocytes/Mfs [193]. In addition, in vitro CD146+ TSCs when primed with IL-1β and CTGF displayed a higher expression of IL-10 that reduced the proinflammatory M1 cells during the tendon healing phase. Curiously, in CTGF delivery tendons, the pro/anti-inflammatory IL-6 showed a temporal expression and appeared reduced by 1 week after surgery [194,195]. Without CTGF, MMP-3 expression was localized on the healing junction and could be associated with matrix degradation, whereas in CTFG treated samples MMP-3 expressions was along with reorienting collagen fibers and could be associated with matrix remodeling. Transcriptional factor STAT-3, that has roles in mediating inflammation, regulating the expression of cytokines such as IL-10 [196,197,198], was significantly upregulated in CTGF treated samples compared with the untreated ones as well as JNK [199] ant TIMP3. Furthermore, the activation of STAT3 is required for IL-10 anti-inflammatory effects and subsequent IL-10 synthesis. This data suggests a novel JNK and STAT3 signaling axis in CTGF-induced IL-10 and TIMP-3 expression of CD146+ TSCs useful to understand the immune regulation in tendon regeneration.

Amniotic epithelial Stem cells (AECs) are recently emerging as a new source of stem cells to be used for tendon regeneration [19,20,200].

Their teno-regenerative role has been rigorously demonstrated by combining in vitro with ex vivo evidence [19,129,200]. The peculiarity of AECs resides in their innate ability to modulate the immune response, contrarily to MSCs that need to be stimulated to induce an efficient effect in term of immunomodulation [166]. The AECs innate immune-regulative properties derives by their physiological role during pregnancy as they mediate the immune interface between the mother and the fetus through the production of cytokines [201].

Ovine AECs (oAECs) demonstrated to have immunomodulatory properties in vitro inhibiting lymphocyte proliferation both in transwell co-culture or in cell-to cell contact systems in vitro [202,203]. Their immunomodulatory effect was tested also in vivo after transplantation in tendon where oAECs inhibited the recruitment of leukocytes mediated by secretion factors and influenced the in-situ macrophages activation and polarization [20]. In particular, the tendon regeneration mediated by oAECs displayed centripetal characterization that begin from the healthy portion of the tendon and progressively invade that wounded part injected with oAECs [19]. In this situation, oAECs migration into the tendon defects seems necessary to drive the tendon healing centripetal progression and M recruitment and activation. M-derived anti-inflammatory cytokines and collagen breakdown enzymes are typically implicated in tissue healing [204]. oAECs transplantation stimulate an anti-inflammatory and antifibrotic mechanism demonstrated also in other tissue such as lung and liver [130,205,206,207], that contribute to M2 polarization thanks to interleukins such as IL-4, IL-10 and IL-13 [208,209] that can be produced by fetal membranes [210,211]. In the study of Mauro et al., after 14 days from oAECs transplantation, a reduced presence of pro-inflammatory M1, usually present during the acute phase of inflammation in response to stimuli such as IFN-g and TNF-α [20,208], was detected. Contextually, the activation of M2 phenotype population was present only in the area actively involved in ECM deposition and remodeling. At day 14, IL-10 expression was higher compared to the other time points and to the control sample, representing a positive repairing effect as IL-10 prevent the production of IL-1, TNF-α, IL-12 and other proinflammatory factors [212,213,214,215]. On day 28, oAECs treated tendons acquired a healthy-like structure with a decrease in M2 phenotypic cells and associated markers, indicating that their pro-regenerative action had ended. Interestingly, control tissue presented a high degree of disorganization and pro-inflammatory M1 markers. The low IL-12β and IL-10 ratio detected in oAEC transplanted tendons, indicated a M shifting towards M2 phenotype [130]. oAECs were able to decrease inflammation during tendon healing promoting M2 polarization and releasing TGF-β and VEGF [19,216] as well as IL-10 influencing MMPs and TIMPs inhibitors activity [217]. Even if the molecular mechanism between oAECs and macrophages during tendon repair remains to be clarified, these results suggest a role of oAECs in guiding the macrophage polarization promoting the regeneration and decreasing inflammation.

**Figure 4:**
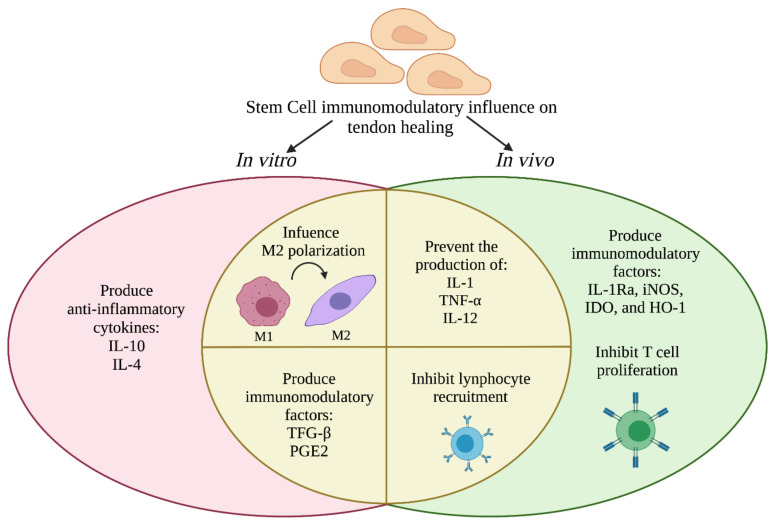
Schematic representation of the prevalent immunomodulatory activities of stem cells during tendon healing tested in vitro and in vivo. The image was adapted from Plock et al. [223].

According to the evidence described above, stem cells such as MSCs or AECs can influence the immune response during tendon healing prevalently modulating the M1 to M2 switch, secreting anti-inflammatory cytokines (IL-4, IL-10, IL-13), preventing the production of pro-inflammatory molecules (iNOS, IDO, TGF-β, PGE_2_) and cytokines, avoiding the proliferation of T cells and recruitment of lymphocytes, as summarized in Figure 4.

Stem cells free regenerative approaches involved bioactive secretomes collected from stem cells as well as hemo-derivate were used in tendon TE [218,219]. Conditioned media (CM) consist in the secretome produced by stem cells and it is composed by multiple factors such as soluble factors and extracellular vesicles [220]. It represent a novel alternative to stem cells therapy that appears to be a promising approach to improve tendon regeneration [219].

In vitro experiment demonstrated the ability of CM collected from oAECs to drive tendon differentiation [221]. Moreover, CM collected from human AEC (hAECs) was proven to have immunomodulatory properties in vitro as it was able to promote the metabolic switch from M1 to M2 [203]. Moreover, a further in vitro study on hAECs CM demonstrated its immunomodulatory properties as it was able to decrease the lymphocyte proliferation [166].

In vivo experiment on 13 horses, demonstrated that tendon healing can be improved by horse AECs CM injection in spontaneous tendon defect [137]. After the confirmation of the immunomodulatory properties of AECs CM obtained in vitro, showing an inhibition of peripheral blood mononuclear cells (PBMCs) proliferation in direct or indirect co-culture, the CM was injected in vivo. The clinical outcomes were encouraging, with the horses experiencing a significantly decreased rate (15.38%) of reinjuries when compared to untreated animals [137].

The efficacy of CM collected from AECs was demonstrated also in comparison with CM collected from MSCs [222]. After injection of the different CM in horse’s tendon lesion, the animals treated with AECs CM displayed a more rapid resuming of activity and a lower rate of re-injury. This evidence demonstrated that conditioned media can be used to improve tendon healing and that the ones collected from AECs can be more effective.

The platelet rich plasma (PRP) therapy was also proven to exhibit a positive effect in tendon healing [224]. In the study of Nishio et al., leukocyte-rich (LR) PRP and leukocyte-poor (LP) PRP were inoculated into mice patellar tendons defect. Results demonstrated that LP-PRP promoted the best tendon healing compared to LR-PRP. It was found that after 4 days of surgery the M1 were higher in both cases, but at day 7 and 14, M2 significantly increased in the LP-PRP group compared to the control groups. PRP improved the tendon healing and stimulated the recruitment of M to the injured tissue. The M typologies differed based on the types of PRPs, implying that leukocytes in PRP alter the efficacy of PRP therapy [224].

The gene therapy can be considered in order to enhance the immunomodulatory properties of stem cells [225,226]. Studies demonstrated that BM-MSCs treated with CRISPR/Cas9 technique to overexpress IL-10, restrict the immune cell accumulation, and pro-inflammatory response in the diabetes-associated myocardial infarction model [227].

Moreover, BM-MSCs genetically modified to overexpress prostaglandin I synthase (PGIS) protected damaged heart and restored cardiac function in a mouse model [228] and modification to enhance therapeutic genes such as IL-4, IL-10, TGF-β1, and GATA-4 demonstrated to increase cell survival and therapeutic effects [229]. Even if this technique has not been used in tendon to date, it could represent a valid approach to modulate the inflammatory response which in turn promote tendon healing and regeneration.

The immunomodulatory properties of stem cells have a fundamental importance in tendon regenerative medicine, but literature lacks on this argument. Certainly, more investigations are needed to deeply comprehend the influence of different stem cells sources on the regenerative process, to choose the suitable type to be used in vivo.

Taking all together, although the promising results obtained with the use of stem cells in vivo in preclinical studies, their translation into the clinic often is difficult to achieve. Considering the complexity and functionality of tendon tissue, the main cause could be due to the fact that the experimental induced tendon injury (i.e., mechanical injury, enzyme and cytokines introduction) does not represent in full a tendinopathy model and this can explain the different outcomes between the preclinical in vivo studies and clinical trials [230]. Additionally, most of the preclinical studies of injured tendons’ models were conducted for a short-term period to verify the underlying mechanisms and the changes occurring within the non-treated and stem cells-treated tendons in contrast to clinical trials that requires a long-term follow up [230]. The different sources of stem cells used for tendon regeneration together with their diverse properties imply to take into consideration different issues that are still not answered including stem cell origin, their transplantation number, the administration technique, and timing [96].

## 7. Conclusions

Inflammation is rapidly becoming recognized as a significant factor in tendon illnesses. Advances in understanding the inflammation’s underlying the cellular mechanisms and the molecular pathways involved have given more insights into tendon illness, as well as the circumstances that may aggravate it, but this information could lead to the development of new therapeutical approaches to be applied for tendinopathies.

Regenerative medicine represents an innovative strategy to deal with tendinopathies and the resulting inflammation. The application of stem cells or their derived secretomes has shown a great potential in improving tendon regeneration allowing to hypothesize the development of cell-based and cell-free strategies to modulate the immune response in tendon injuries. Indeed, it has been demonstrated that different immune cells together with secreted immune modulators participate to control and promote tissue regeneration. Understanding the crosstalk between the host tissue, stem and immune cells might greatly modulate the immune reaction resolving hence the inflammatory response and avoiding the formation of fibrotic scar tissue. Therefore, a prolonged inflammation could be avoided by modulating the immune cells either with bioactive immunomodulating molecules, such as the stem cells’ secretomes, or by directly applying stem cells with an immunomodulatory potential, as AECs, which in turn might lead to an improved tendon regeneration. Encouraging prospects in this field are represented by stimulating whether macrophage polarization towards anti-inflammatory phenotype whether stem cell recruitment and penetration within the damage tissue accompanied with an enhancement of their immunomodulatory properties. Taking together, understanding the interplay between different tendon cell compartments together with the potential role of stem cells in modulating the inflammatory response due to their immunomodulatory properties helps to develop new innovative strategies to deal with tendinopathies. Evaluating the immune-related mechanisms within healthy and injured tendon tissues serves to clearly assess the different inflammatory processes for further targeted strategies to deal with tendinopathies.

## Figures and Tables

**Table 1:** DAMPs and their implications in tendinopathy.

DAMPs	Receptors	Biological Activity	Reference
HMGB1	RAGE, TLR-2, TLR-4, TLR-9	↑ Pro-inflammatory cytokines and stromal cell compartments responses	[3,72]
HSPs	TLR-4	Cytokine and chemokine release and activation of NK cells	[74]
S100A8/A9	TLR-4	Attraction of T cells, neutrophils, and macrophages	[73]

HMGB1: high-mobility group box protein 1; HSPs: heat shock proteins; RAGE: receptor for advanced glycation end products; TLR-2, -4, -9: toll-like receptor-2, -4, -9; ↑: increase

## Data Availability

Not applicable.
